# Serum liver fibrosis markers predict hepatic decompensation in compensated cirrhosis

**DOI:** 10.1186/s12876-023-02877-2

**Published:** 2023-09-19

**Authors:** Qingling Chen, Ling Mei, Rui Zhong, Ping Han, Jun Wen, Xu Han, Lu Zhai, Lili Zhao, Jia Li

**Affiliations:** 1 Clinical School of the Second People’s Hospital, Tianjin Medical University, Tianjin, China; 2 Department of Gastroenterology and Hepatology, Tianjin Second People’s Hospital, No.7, Sudi South Road, Nankai District, Tianjin, 300192 China; 3 Department of Neurology, The First Hospital of Jilin University, Changchun, Jilin China

**Keywords:** Biomarker, Fibrosis, Compensated cirrhosis, Hepatic decompensation, Prognosis

## Abstract

**Background and aim:**

The literature is sparse on the association between serum liver fibrosis markers and the development of hepatic decompensation in patients with compensated cirrhosis. We aimed to assessed whether the serum liver fibrosis markers are predictive of the occurrence of hepatic decompensation.

**Methods:**

We ascertained 688 cirrhotic patients with varying etiologies, between December 2015 to December 2019. Serum hyaluronic acid (HA), laminin (LN), collagen IV (CIV), and N-terminal propeptide of type III collagen (PIIINP) levels were measured at enrollment. All subjects were followed for at least 6 months for occurrence of hepatic decompensation. Cox proportional hazard regression models were used to estimate the hazard ratios (HRs) of hepatic decompensation during follow-up.

**Results:**

During a median follow-up of 22.0 (13.0–32.0) months, decompensation occurred in 69 (10.0%) patients. Multivariate analysis indicated that higher LN (HR: 1.008, 95% confidence interval [CI]: 1.002–1.014, *P* = 0.011) and CIV (HR: 1.004, 95% CI: 1.001–1.007, *P* = 0.003) levels were independently associated with hepatic decompensation. Furthermore, patients in the tertile 2 and tertile 3 groups for CIV levels had HRs of 4.787 (1.419, 16.152) (*P* = 0.012) and 5.153 (1.508, 17.604) (*P* = 0.009), respectively, for occurrence of decompensation event compared with those in the tertile 1 group.

**Conclusion:**

Serum liver fibrosis markers, particularly in CIV, appeared to be reliable biomarkers of disease progression and liver decompensation in patients with compensated cirrhosis with varying etiologies.

## Introduction

The natural history of chronic liver disease is characterized by the progression to cirrhosis, first compensated, and then decompensated, which is associated with high mortality [1]. Hepatic decompensation is defined as the development of ascites, variceal bleeding, or hepatic encephalopathy (HE) [2]. Identification of predictors of decompensation among compensated patients is warranted because death in cirrhosis is clearly related to the development of decompensation. One of the main predictors of outcome is the stage of liver fibrosis in chronic liver disease [3]. Liver biopsy remains the gold standard method for the assessment of liver fibrosis. However, the clinical application of liver biopsy is often limited by its invasiveness, high cost, sampling variability, interobserver variation, risk of complications, and poor patient compliance, particularly in the follow-up period [4–6]. Therefore, it is desirable to develop and verify noninvasive and convenient markers to accurately evaluate liver fibrosis and inform the prognosis of liver disease.

Advances in serological and radiological tests such as serum markers, transient elastography, and their combination can achieve accurate evaluation of fibrosis and reduce the need for liver biopsy. Because hepatic fibrosis is characterized by the excessive deposition of extracellular matrix (ECM), serum markers representing ECM components are widely employed to assess the development of hepatic fibrosis [7, 8]. Hyaluronic acid (HA), laminin (LN), collagen IV (CIV), and N-terminal propeptide of type III collagen (PIIINP) are four major serum markers for the non-invasive assessment of liver fibrosis. So far, numerous studies have shown their potential clinical value in the diagnosis of liver fibrosis and cirrhosis [9–14]. Theoretically, the stage of liver fibrosis is positively correlated with the severity of liver dysfunction, which affects the survival conditions. Plevris N et al. [15]reported that HA measurement can accurately and independently predict liver-related and all‐cause mortality in patients with liver disease. However, few studies have explored the clinical significance of HA, LN, CIV, and PIIINP in patients with liver cirrhosis. Moreover, it remains unclear whether these four serum liver fibrosis markers can predict the prognosis of patients with compensated liver cirrhosis.

Currently available survival scoring systems, such as the Child–Pugh score (CPS) and the model for end-stage liver disease (MELD) score, have been widely validated as accurate predictors of short or medium-term survival in patients with liver cirrhosis [2]. However, their accuracy is limited in compensated cirrhosis and non‐cirrhotic liver disease because they depend on variables reflecting pathophysiological changes associated with advanced disease [16]. Early identification of cirrhotic patients at risk for hepatic decompensation remains a major challenge. Thus, we assessed whether the HA, LN, CIV, and PIIINP levels are non-invasive predictors of hepatic decompensation in patients with compensated cirrhosis with varying etiologies.

## Materials and methods

### Study population

We conducted a retrospective cohort analysis of the electronic medical record database of all consecutive cases of patients diagnosed with compensated cirrhosis at the Department of Gastroenterology and Hepatology, Tianjin Second People’s Hospital, Tianjin, China, from December 2015 to December 2019. The diagnosis of cirrhosis was based on the typical imaging features, histological features, and/or presence of varices on endoscopy. Patients with previous liver decompensation (ascites, variceal bleed and HE), acute-on-chronic liver failure, or liver transplantation, patients with a known diagnosis of hepatocellular carcinoma, patients with severe extrahepatic diseases with poor short-term prognosis, or follow-up < 6 months were excluded from this study.

This study protocol was reviewed and approved by the Ethics Committee of Tianjin Second People’s Hospital, approval number [JINERRENMINLUNSHENZI (2021) NO.54], and conducted according to the principles of the Declaration of Helsinki. All patients signed a written informed consent document and gave permission for the clinical and laboratory data for study purposes.

### Baseline evaluation

Patients’ demographic, clinical, and laboratory profiles at admission were recorded from the hospital electronic clinical records system. The following variables were confirmed: age, gender, etiology of liver cirrhosis, esophageal varices, received antiviral therapy, and laboratory data (alanine aminotransferase [ALT], aspartate aminotransferase [AST], alkaline phosphatase [ALP], gamma-glutamyl transpeptidase [GGT], albumin [ALB], total bilirubin [TBIL], serum creatinine [Cr], international normalised ratio [INR], and platelet count [PLT]). For all patients, blood samples were collected on the same day. Serum ALT, AST, ALP, GGT, ALB, TBIL and Cr were detected by a Hitachi 7180 Automatic Biochemical Analyser (Hitachi, Ltd, Tokyo, Japan). The MELD score, fibrosis-4 score (FIB-4) index, and AST-to-PLT ratio index (APRI) were calculated for each patient utilising laboratory data. The FIB-4 was calculated with the equation FIB-4 = [Age (years) * AST (U/L)]/[PLT (10^9^/L) * ALT (U/L) ^ (1/2)] [17]. The APRI was calculated with the equation APRI = 100 × (AST / ULN) / (PLT (×10^9^/L) [18]. Architect chemiluminescence analyzer was used to detect HBsAg and hepatitis B e antigen (HBeAg). The lower limit of quantification of quantitative HBsAg levels was 0.05 IU/mL, and that of quantitative HBeAg levels was 1.0 S/CO (≥ 1.0 S/CO indicates positive HBeAg). Serum hepatitis B virus deoxyribonucleic acid levels were assayed by automatic real-time fluorescent quantitative polymerase chain reaction technique (Cobas Taqman; Roche Diagnostics, GmbH, Mannheim, Germany), with the lowest detection limit of 20 IU/mL; the values were log transformed with units expressed in log _10_ IU/mL. The coagulation tests were performed by the clotting method on the automatic coagulometer STAGO Compact (Diagnostica Stago, France). The complete blood count was measured using a Sysmex XN-2000 haematology analyser (Sysmex corporation, Kobe, Japan) according to the manufacturer’s recommendation. All cirrhotic patients underwent upper gastrointestinal endoscopy for variceal screening at baseline. Esophageal varices were graded as none, small (<5 mm diameter) or large (≥ 5 mm diameter) according to the Baveno VI guidelines [19].

### Serum liver fibrosis markers

All patients underwent baseline detection of serum liver fibrosis markers including HA, LN, CIV, and PIIINP to stage the liver disease at the discretion of their attending clinician. Blood samples drawn between 8:00 and 11:00 am after overnight fasting were used for the assays. The levels of HA, LN, CIV, and PIIINP were detected by magnetic microparticle-based chemiluminescent immunoassays using the AutoLumo A2000 Plus Fully Automated Chemiluminescence Immunoassay System (Autobio Diagnostics Co., Ltd, Zhengzhou, China). The reference values were: HA < 120 ng/mL, LN < 130 ng/mL, CIV < 95 ng/mL, and PIIINP < 15 ng/mL. The intra-assay and interassay coefficients of variation were ≤ 15.0% for the four serum liver fibrosis markers.

### Follow-up and study outcomes

Usually, patients were evaluated after every 3–6 months for the presence of hepatic decompensation and for the development of hepatocellular carcinoma. The follow-up started from the inclusion of the study and ended in January 2022. The clinical outcomes were carefully recorded. The outcome of hepatic decompensation was defined as the occurrence of liver-related complications, such as ascites, variceal bleeding, or HE [20]. Ascites was defined as the development of de novo ascites requiring initiation of diuretic therapy. Variceal bleeding was defined as portal hypertension related variceal bleeding requiring hospital admission [19]. HE was defined as the development of grade P2 HE requiring hospital admission [21]. Severity of liver disease was assessed by the MELD score.

The primary outcome was the nature and time of the first hepatic decompensation event. The end point was decompensation-free survival. We have combined liver decompensation and death for analysis because most deaths are attributable to decompensation in advanced liver disease. We also assessed the rate and predictors of hepatic decompensation.

### Statistical analysis

Continuous variables are expressed as median with interquartile range and compared using the Mann–Whitney U test. Categorical variables are presented as frequency and percentage and were compared using Chi-square test. The number (proportion) of development of hepatic decompensation according to the tertiles of serum liver fibrosis markers were also compared using the Cochran-Armitage trend test. Outcomes were analyzed as time-to-event variables. In these analyses, the cumulative incidence function of the analyzed events was estimated. The univariate and multivariate Cox regression models were performed to investigate the influence of serum liver fibrosis markers on hepatic decompensation during follow-up. Multivariate Cox regression models were created including the variables associated with the outcome in the univariate analysis with *P* < 0.05. The univariate and multivariate Cox regression models were also employed to assess the hazard ratios (HR) predicting the presence of hepatic decompensation with the increasing tertile of the serum liver fibrosis markers levels. In these Cox regression models, decompensating events during follow-up were evaluated as time-dependent variables. Survival curves were compared between groups using the Kaplan–Meier method with log-rank tests. All *P* values were 2-tailed and values < 0.05 were considered statistically significant. Statistical analyses were performed by SPSS Statistics 26 (IBM, New York, NY, USA). The survival curve was drawn in the survival and survminer packages in R version 4.2.2 (R Foundation for Statistical Computing, Vienna, Austria) using the post-hoc Bonferroni method.

## Results

### Baseline characteristics

A total of 688 consecutive patients with compensated cirrhosis were evaluated. The median age was 52.0 (41.0–59.0) years, and 59.9% of patients were males. Hepatitis B virus -induced cirrhosis was the most common (70.6%), followed by hepatitis C (13.8%), autoimmune (4.8%), and alcoholic cirrhosis (3.2%). The remaining (7.6%) patients were classified as other types of liver cirrhosis. In the overall series, median serum HA, LN, CIV, and PIIINP levels were 134.5 (93.0-295.3) ng/mL, 95.0 (85.0-116.0) ng/mL, 72.0 (62.0-133.0) ng/mL, and 9.7 (7.0–14.0) ng/mL, respectively. The median MELD score, APRI, and FIB-4 were 7.0, 0.94, and 2.72, respectively. Further information on the characteristics of the study subjects is summarized in Table 1.

**Table 1:** Baseline demographic, clinical, and laboratory profiles based on the development of hepatic decompensation during follow-up

Variable	Overall (n = 688)	No decompensation (n = 619)	Decompensation (n = 69)	*P* value
Age (years)	52.0 (41.0–59.0)	51.0 (40.0–58.0)	55.0 (49.0–62.0)	< 0.001
Males, n (%)	412 (59.9)	374 (60.4)	38 (55.1)	0.390
Etiology of cirrhosis, n (%)				0.001
Hepatitis B	486 (70.6)	451 (72.9)	35 (50.7)	
Hepatitis C	95 (13.8)	83 (13.4)	12 (17.4)	
Autoimmune	33 (4.8)	29 (4.7)	4 (5.8)	
Alcohol	22 (3.2)	15 (2.4)	7 (10.1)	
Others	52 (7.6)	41 (6.6)	11 (15.9)	
Esophageal varices, n (%)				< 0.001
None	409 (59.4)	396 (64.0)	14 (20.3)	
Small	279 (40.6)	223 (36.0)	55 (79.7)	
ALT (U/L)	45.0 (24.0-109.0)	46.0 (24.0-116.0)	31.0 (21.0–60.0)	0.007
AST (U/L)	42.0 (26.0–90.0)	43.0 (25.0–96.0)	39.0 (28.5–66.0)	0.656
ALP (U/L)	77.0 (60.0-101.8)	75.0 (59.0–98.0)	93.0 (73.5–119.0)	< 0.001
GGT (U/L)	59.0 (32.0-120.8)	57.0 (31.0-117.0)	76.0 (42.0-178.5)	0.014
ALB (g/L)	43.0 (39.1–46.2)	43.3 (39.5–46.4)	39.6 (36.0-42.6)	< 0.001
TBIL (μmol/L)	17.4 (13.3–24.4)	17.2 (13.2–23.8)	19.2 (13.9–33.0)	0.018
Cr (μmol/L)	59.5 (50.0–70.0)	60.0 (51.0–70.0)	54.0 (47.0-64.5)	0.012
INR	1.06 (1.00-1.15)	1.06 (1.00-1.14)	1.14 (1.05–1.31)	< 0.001
PLT (×10^9^/L)	133.0 (96.0-172.0)	140.0 (102.0-176.0)	87.0 (56.5-114.5)	< 0.001
MELD score	7.0 (6.0–9.0)	7.0 (6.0–9.0)	8.0 (7.0–11.0)	< 0.001
APRI	0.94 (0.48–2.12)	0.89 (0.46–2.05)	1.42 (0.85–2.44)	0.003
FIB-4 index	2.72 (1.54–4.97)	2.48 (1.47–4.65)	4.61 (3.49–7.79)	< 0.001
Serum liver fibrosis markers				
HA (ng/mL)	134.5 (93.0-295.3)	127.0 (93.0-271.0)	257.0 (122.0-459.5)	< 0.001
LN (ng/mL)	95.0 (85.0-116.0)	94.0 (84.0-110.0)	112.0 (92.5-165.5)	< 0.001
CIV (ng/mL)	72.0 (62.0-133.0)	69.0 (62.0-125.0)	138.0 (83.5–199.0)	< 0.001
PIIINP (ng/mL)	9.7 (7.0–14.0)	9.7 (7.0–14.0)	11.0 (8.4–16.0)	0.077
Length of follow-up (months)	22.0 (13.0–32.0)	22.0 (13.0–31.0)	20.0 (10.5–35.5)	0.705
hepatic decompensation, n (%)	69 (10.0)			
Ascites, n (%)	56 (8.1)			
Variceal bleeding, n (%)	27 (3.9)			
Hepatic encephalopathy, n (%)	14 (2.0)			

Data are expressed as median (interquartile range) or frequency (percentage) where appropriate

ALT, alanine aminotransferase; AST, aspartate aminotransferase; ALP, alkaline phosphatase; GGT, gamma-glutamyl transpeptidase; ALB, albumin; TBIL, total bilirubin; Cr, Creatinine; INR, international normalized ratio; PLT, platelet; MELD, model for end-stage liver disease; APRI, AST-to-PLT ratio index; FIB-4, fibrosis-4 score; HA, hyaluronic acid; LN, laminin; CIV, collagen IV; PIIINP, N-terminal propeptide of type III collagen

### Follow-up

**Fig. 1:**
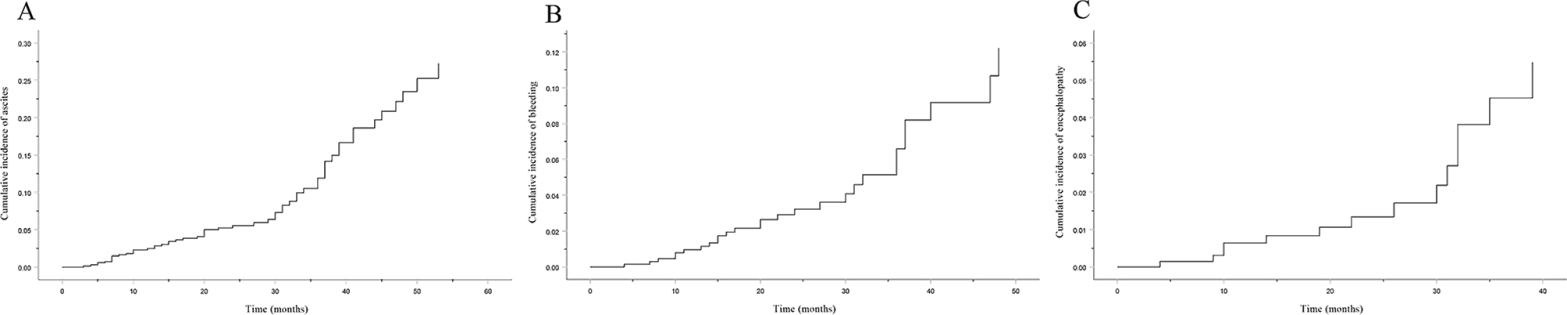
Cumulative incidence of liver decompensation in patients with compensated cirrhosis. During a median follow-up of 22.0 (13.0–32.0) months, a total of 69 patients developed hepatic decompensation. Among them, 56 patients developed ascites, 27 patients developed variceal bleeding, and 14 patients developed HE. (**A**) The cumulative incidence of ascites. (**B**) The cumulative incidence of variceal bleeding. (**C**) The cumulative incidence of HE. HE, hepatic encephalopathy

Table 1 shows the median length of follow-up was 22.0 (13.0–32.0) months. A total of 69 (10.0%) patients developed hepatic decompensation during follow-up. Ascites (n = 56, 8.1%) was the most common decompensating event, followed by variceal bleeding (n = 27, 3.9%) and HE (n = 14, 2.0%). Figure 1 depicts the cumulative incidence of liver decompensation in these patients with compensated cirrhosis. Obviously, ascites was the most frequent decompensating events occurring in this cohort of patients. We compared baseline demographic, clinical, and laboratory profiles between patients developing hepatic decompensation group and without development of hepatic decompensation group during follow-up. Patients developing hepatic decompensation had significantly higher serum HA, LN, and CIV levels compared to those patients who did not develop hepatic decompensation (257.0 [122.0-459.5] vs. 127.0 [93.0-271.0] ng/mL, 112.0 [92.5-165.5] vs. 94.0 [84.0-110.0] ng/mL, and 138.0 [83.5–199.0] vs. 69.0 [62.0-125.0] ng/mL, respectively, all *P* < 0.001). Although no significant differences in PIIINP levels were found between the two groups, the *P* value was close to 0.05 (*P* = 0.077). In addition, the age, ALP, GGT, TBIL, INR, MELD score, APRI, and FIB-4 index were higher among patients developing hepatic decompensation than among those without development of hepatic decompensation (*P*<0.05). The proportion of alcoholic cirrhosis and small esophageal varices were higher in patients developing hepatic decompensation than in those who did not develop hepatic decompensation (*P*<0.05). By contrast, a trend for lower ALT, ALB, Cr, and PLT were observed in patients who developed hepatic decompensation (*P*<0.05).

### Baseline tertiles of serum liver fibrosis markers and the development of hepatic decompensation during follow-up

The patients were divided into three subgroups based on tertiles of serum liver fibrosis markers levels. The number (proportion) of development of hepatic decompensation according to the tertiles of serum liver fibrosis markers are presented in Table 2. The proportion of hepatic decompensation increased as the serum HA, LN, CIV, and PIIINP levels increased from tertile 1 to tertile 3 (*P*<0.001, *P*<0.001, *P*<0.001, and *P* = 0.014, respectively).

**Table 2:** Baseline tertiles of serum liver fibrosis markers based on the development of hepatic decompensation during follow-up

Serum liver fibrosis markers	Range	No decompensation, (n = 619)	Decompensation, (n = 69)	*P* value
HA	(ng/mL)			< 0.001
Tertile 1, n = 229	30.0–97.0	220 (35.5)	9 (13.0)	
Tertile 2, n = 229	97.0-220.0	209 (33.8)	20 (29.0)	
Tertile 3, n = 230	220.0-6287.0	190 (30.7)	40 (58.0)	
LN	(ng/mL)			< 0.001
Tertile 1, n = 229	15.0–91.0	213 (34.4)	16 (23.2)	
Tertile 2, n = 229	91.0-100.0	216 (34.9)	13 (18.8)	
Tertile 3, n = 230	100.0-353.0	190 (30.7)	40 (58.0)	
CIV	(ng/mL)			< 0.001
Tertile 1, n = 229	10.0–65.0	226 (36.5)	3 (4.3)	
Tertile 2, n = 229	65.0-108.0	205 (33.1)	24 (34.8)	
Tertile 3, n = 230	108.0-612.0	188 (30.4)	42 (60.9)	
PIIINP	(ng/mL)			0.014
Tertile 1, n = 229	2.0–8.0	216 (34.9)	13 (18.8)	
Tertile 2, n = 229	8.0–12.0	202 (32.6)	27 (39.1)	
Tertile 3, n = 230	12.0–61.0	201 (32.5)	29 (42.0)	

Data are expressed as frequency (percentage)

HA, hyaluronic acid; LN, laminin; CIV, collagen IV; PIIINP, N-terminal propeptide of type III collagen

### Factors associated with hepatic decompensation

We used both univariate and multivariate Cox regression analyses to identify the indicators related to hepatic decompensation. In the univariate Cox regression analysis, the factors associated with hepatic decompensation were etiology of alcoholic cirrhosis (HR: 4.309, 95% confidence interval [CI]: 1.911–9.715, *P* < 0.001), small esophageal varices (HR: 5.143, 95% CI: 2.858–9.256, *P* < 0.001), older age (HR: 1.038, 95% CI: 1.014–1.062, *P* = 0.001), higher ALP (HR: 1.003, 95% CI: 1.001–1.004, *P* = 0.003), GGT (HR: 1.001, 95% CI: 1.001–1.002, *P* < 0.001), MELD score (HR: 1.098, 95% CI: 1.047–1.151, *P* < 0.001), FIB-4 index (HR: 1.086, 95% CI: 1.050–1.124, *P* < 0.001), LN (HR: 1.015, 95% CI: 1.011–1.020, *P* < 0.001) and CIV (HR: 1.007, 95% CI: 1.005–1.009, *P* < 0.001) levels, and lower ALB level (HR: 0.914, 95% CI: 0.879–0.950, *P* < 0.001) and PLT counts (HR: 0.983, 95% CI: 0.977–0.988, *P* < 0.001) at baseline. After adjusting for potential confounders using a multivariable Cox regression model, etiology of alcoholic cirrhosis (HR: 2.447, 95% CI: 1.018–5.883, *P* = 0.046), small esophageal varices (HR: 3.341, 95% CI: 1.770–6.306, *P* < 0.001), older age (HR: 1.042, 95% CI: 1.013–1.072, *P* = 0.005), higher GGT (HR: 1.001, 95% CI: 1.001–1.002, *P* = 0.004), LN (HR: 1.008, 95% CI: 1.002–1.014, *P* = 0.011) and CIV (HR: 1.004, 95% CI: 1.001–1.007, *P* = 0.003) levels, and lower PLT counts (HR: 0.985, 95% CI: 0.978–0.992, *P* < 0.001) were found to be independently associated with hepatic decompensation (Table 3).

**Table 3:** Univariate and multivariate analysis for baseline predictors of hepatic decompensation

Variable	Univariate analysis		Multivariate analysis	
	HR (95% CI)	*P* value	HR (95% CI)	*P* value
Age	1.038 (1.014–1.062)	0.001	1.042 (1.013–1.072)	0.005
Males	0.820 (0.510–1.319)	0.414		
Etiology of cirrhosis				
Hepatitis B	Reference		Reference	
Hepatitis C	1.895 (0.983–3.652)	0.056	1.433 (0.706, 2.906)	0.319
Autoimmune	1.662 (0.591–4.680)	0.336	1.732 (0.540, 5.553)	0.356
Alcohol	4.309 (1.911–9.715)	< 0.001	2.447 (1.018, 5.883)	0.046
Others	3.040 (1.543–5.988)	0.001	3.601 (1.709, 7.586)	0.001
Small esophageal varices	5.143 (2.858, 9.256)	< 0.001	3.341 (1.770, 6.306)	< 0.001
ALT	0.999 (0.997–1.001)	0.219		
AST	1.000 (0.998–1.001)	0.723		
ALP	1.003 (1.001–1.004)	0.003	1.001 (0.997–1.006)	0.607
GGT	1.001 (1.001–1.002)	< 0.001	1.001 (1.001–1.002)	0.004
ALB	0.914 (0.879–0.950)	< 0.001	0.981 (0.936–1.028)	0.420
TBIL	1.003 (0.997–1.009)	0.348		
Cr	0.986 (0.969–1.003)	0.113		
INR	1.128 (0.946–1.346)	0.180		
PLT	0.983 (0.977–0.988)	< 0.001	0.985 (0.978–0.992)	< 0.001
MELD score	1.098 (1.047–1.151)	< 0.001	0.989 (0.901–1.085)	0.813
APRI	1.034 (0.998–1.072)	0.067		
FIB-4 index	1.086 (1.050–1.124)	< 0.001	0.934 (0.857–1.018)	0.119
HA	1.000 (1.000-1.001)	0.591		
LN	1.015 (1.011–1.020)	< 0.001	1.008 (1.002–1.014)	0.011
CIV	1.007 (1.005–1.009)	< 0.001	1.004 (1.001–1.007)	0.003
PIIINP	1.023 (0.996–1.052)	0.100		

ALT, alanine aminotransferase; AST, aspartate aminotransferase; ALP, alkaline phosphatase; GGT, gamma-glutamyl transpeptidase; ALB, albumin; TBIL, total bilirubin; Cr, Creatinine; INR, international normalized ratio; PLT, platelet; MELD, model for end-stage liver disease; APRI, AST-to-PLT ratio index; FIB-4, fibrosis-4 score; HA, hyaluronic acid; LN, laminin; CIV, collagen IV; PIIINP, N-terminal propeptide of type III collagen; HR, hazard ratio; CI, confidence interval

In addition, we evaluated the predictive factors of decompensation in HBV-related cirrhosis because hepatitis B patients account for the highest proportion in this cohort. We found that after adjusting for potential confounders using a multivariable Cox regression model, higher LN (HR: 1.012, 95% CI: 1.002–1.022, *P* = 0.015) and CIV (HR: 1.005, 95% CI: 1.001–1.008, *P* = 0.024) levels were still independently associated with hepatic decompensation (Table 4).

**Table 4:** Univariate and multivariate analysis for baseline predictors of hepatic decompensation in HBV-related cirrhosis

Variable	Univariate analysis		Multivariate analysis	
	HR (95% CI)	*P* value	HR (95% CI)	*P* value
Age	1.046 (1.014–1.080)	0.005	1.054 (1.015–1.095)	0.007
Males	1.355 (0.693–2.649)	0.375		
Small esophageal varices	6.424 (2.660-15.513)	< 0.001	2.855 (1.139–7.158)	0.025
ALT	0.992 (0.985-1.000)	0.054		
AST	0.994 (0.988–1.001)	0.111		
HBV DNA	0.973 (0.836–1.133)	0.727		
qHBsAg (× 10^3^)	0.810 (0.672–0.977)	0.027	0.883 (0.731–1.066)	0.196
HBeAg positive	1.044 (0.530–2.056)	0.901		
Received antiviral therapy	1.213 (0.166–8.873)	0.849		
ALB	0.899 (0.852–0.948)	< 0.001	1.032 (0.956–1.113)	0.423
TBIL	1.006 (0.992–1.020)	0.431		
Cr	0.988 (0.964–1.013)	0.346		
PLT	0.972 (0.963–0.981)	< 0.001	0.975 (0.962–0.988)	< 0.001
MELD score	1.167 (1.064–1.281)	0.001	0.946 (0.816–1.095)	0.456
APRI	0.988 (0.884–1.104)	0.830		
FIB-4 index	1.131 (1.073–1.192)	< 0.001	0.929 (0.803–1.075)	0.322
HA	1.000 (1.000-1.001)	0.621		
LN	1.016 (1.009–1.022)	< 0.001	1.012 (1.002–1.022)	0.015
CIV	1.007 (1.004–1.009)	< 0.001	1.005 (1.001–1.008)	0.024
PIIINP	1.018 (0.980–1.057)	0.362		

ALT, alanine aminotransferase; AST, aspartate aminotransferase; qHBsAg, quantitative hepatitis B surface antigen; HBeAg, hepatitis B e antigen; ALB, albumin; TBIL, total bilirubin; Cr, Creatinine; PLT, platelet; MELD, model for end-stage liver disease; APRI, AST-to-PLT ratio index; FIB-4, fibrosis-4 score; HA, hyaluronic acid; LN, laminin; CIV, collagen IV; PIIINP, N-terminal propeptide of type III collagen; HR, hazard ratio; CI, confidence interval

We also evaluated the predictive factors for ascites due to the highest proportion of patients developing ascites in this cohort. We found that after adjusting for potential confounders using a multivariable Cox regression model, higher LN (HR: 1.010, 95% CI: 1.003–1.017, *P* = 0.007) and CIV (HR: 1.004, 95% CI: 1.001–1.008, *P* = 0.022) levels were also independently associated with ascites (Table 5).

**Table 5:** Univariate and multivariate analysis for baseline predictors of ascites

Variable	Univariate analysis		Multivariate analysis	
	HR (95% CI)	*P* value	HR (95% CI)	*P* value
Age	1.043 (1.017–1.070)	0.001	1.047 (1.011–1.084)	0.011
Males	0.574 (0.339–0.972)	0.039	0.459 (0.235–0.899)	0.023
Etiology of cirrhosis				
Hepatitis B	Reference		Reference	
Hepatitis C	1.839 (0.864–3.912)	0.114	1.185 (0.518–2.710)	0.688
Autoimmune	2.136 (0.747–6.109)	0.157	1.583 (0.483–5.186)	0.448
Alcohol	4.679 (1.928–11.354)	0.001	3.635 (1.340–9.857)	0.011
Others	3.624 (1.753–7.494)	0.001	4.832 (2.184–10.687)	< 0.001
Small esophageal varices	3.879 (2.116, 7.110)	< 0.001	3.098 (1.609, 5.965)	0.001
ALT	0.999 (0.998–1.001)	0.391		
AST	1.000 (0.999–1.001)	0.996		
GGT	1.001 (1.001–1.002)	< 0.001	1.002 (1.001–1.003)	0.001
ALB	0.924 (0.885–0.965)	< 0.001	1.007 (0.954–1.063)	0.804
TBIL	1.003 (0.997–1.010)	0.329		
Cr	0.986 (0.967–1.006)	0.164		
INR	1.135 (0.939–1.373)	0.190		
PLT	0.984 (0.978–0.990)	< 0.001	0.986 (0.977–0.995)	0.002
MELD score	1.101 (1.045–1.160)	< 0.001	1.010 (0.907–1.124)	0.858
APRI	1.039 (1.001–1.079)	0.044	1.037 (0.960–1.121)	0.353
FIB-4 index	1.088 (1.048–1.131)	< 0.001	0.902 (0.789–1.031)	0.130
HA	1.000 (1.000-1.001)	0.560		
LN	1.016 (1.011–1.021)	< 0.001	1.010 (1.003–1.017)	0.007
CIV	1.007 (1.005–1.009)	< 0.001	1.004 (1.001–1.008)	0.022
PIIINP	1.018 (0.986–1.052)	0.275		

ALT, alanine aminotransferase; AST, aspartate aminotransferase; GGT, gamma-glutamyl transpeptidase; ALB, albumin; TBIL, total bilirubin; Cr, Creatinine; INR, international normalized ratio; PLT, platelet; MELD, model for end-stage liver disease; APRI, AST-to-PLT ratio index; FIB-4, fibrosis-4 score; HA, hyaluronic acid; LN, laminin; CIV, collagen IV; PIIINP, N-terminal propeptide of type III collagen; HR, hazard ratio; CI, confidence interval

### Influence of serum liver fibrosis markers on the risk of hepatic decompensation

**Fig. 2:**
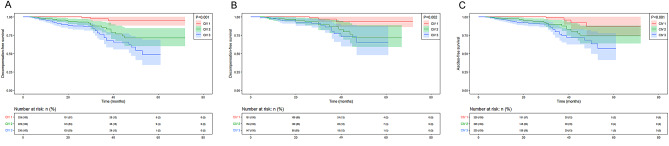
Kaplan-Meier curves showing decompensation-free survival during the follow-up period. (**A**) Decompensation-free survival in all patients categorized according to the tertiles of serum CIV in the whole series (log-rank *P* < 0.001). (**B**) Decompensation-free survival in patients with HBV-related cirrhosis categorized according to the tertiles of serum CIV. (log-rank *P* = 0.002). (**C**) Ascites -free survival in all patients categorized according to the tertiles of serum CIV in the whole series (log-rank *P* < 0.001). CIV, collagen IV.

Based on baseline tertiles of serum liver fibrosis markers, univariate Cox regression analysis were performed and multivariate Cox regression models were created after controlling potential confounding variables, including age, etiology of cirrhosis, small esophageal varices, ALB, PLT, MELD score, and FIB-4 index (Table 6). The rates of hepatic decompensation increased with increasing CIV level. The HR (95% CI) in tertile 2 and tertile 3 were 4.787 (1.419, 16.152) (*P* = 0.012), and 5.153 (1.508, 17.604) (*P* = 0.009), respectively. However, no statistically significant associations were observed between the increasing HA, LN, and PIIINP levels and the rates of hepatic decompensation in multivariable Cox regression models. Figure 2 A shows the decompensation-free survival in all patients categorized according to the tertiles of serum CIV in the whole series. We compared decompensation-free survival by the Kaplan–Meier method in the three groups. The probability of decompensation-free survival decreased with increasing CIV level (log-rank *P* < 0.001). Figure 2B shows the decompensation-free survival in patients with HBV-related cirrhosis categorized according to the tertiles of serum CIV. We also compared decompensation-free survival by the Kaplan–Meier method in the three groups. The probability of decompensation-free survival still decreased with increasing CIV level (log-rank *P* = 0.002). Figure 2 C shows the ascites -free survival in all patients categorized according to the tertiles of serum CIV in the whole series. We compared ascites -free survival by the Kaplan–Meier method in the three groups. The probability of ascites-free survival decreased with increasing CIV level (log-rank *P* < 0.001).

**Table 6:** Univariate and multivariate analysis for baseline tertiles of serum liver fibrosis markers predicting hepatic decompensation

Serum liver fibrosis markers	Unadjusted		Adjusted*	
	HR (95% CI)	*P* value	HR (95% CI)	*P* value
HA (ng/mL)				
Tertile 1	Reference		Reference	
Tertile 2	2.138 (0.973, 4.697)	0.058	1.779 (0.793, 3.993)	0.162
Tertile 3	3.825 (1.855, 7.891)	< 0.001	1.462 (0.682, 3.137)	0.329
LN (ng/mL)				
Tertile 1	Reference		Reference	
Tertile 2	0.838 (0.403, 1.743)	0.636	0.987 (0.466, 2.092)	0.972
Tertile 3	3.042 (1.701, 5.440)	< 0.001	1.659 (0.878, 3.136)	0.119
CIV (ng/mL)				
Tertile 1	Reference		Reference	
Tertile 2	7.367 (2.218, 24.473)	0.001	4.787 (1.419, 16.152)	0.012
Tertile 3	13.496 (4.183, 43.544)	< 0.001	5.153 (1.508, 17.604)	0.009
PIIINP (ng/mL)				
Tertile 1	Reference		Reference	
Tertile 2	2.223 (1.147, 4.310)	0.018	1.368 (0.692, 2.703)	0.367
Tertile 3	2.507 (1.302, 4.827)	0.006	1.287 (0.650, 2.548)	0.469

* Adjustments for age, etiology of cirrhosis, small esophageal varices, ALB, PLT, MELD score, and FIB-4 index

HA, hyaluronic acid; LN, laminin; CIV, collagen IV; PIIINP, N-terminal propeptide of type III collagen; ALB, albumin; PLT, platelet; MELD, model for end-stage liver disease; FIB-4, fibrosis-4 score; HR, hazard ratio; CI, confidence interval

## Discussion

We conducted this large retrospective cohort study to describe the decompensation-free survival and determine the prognostic value of noninvasive serum markers including HA, LN, CIV, and PIIINP in compensated cirrhotic patients at a tertiary medical center in North China. Our results have shown that cirrhotic patients having higher serum HA, LN, CIV, and PIIINP levels have a significantly higher incidence of hepatic decompensation compared with cirrhotic patients with lower serum levels. Using Cox regression models, we have also shown that serum LN and CIV levels predict occurrence of decompensation event independent of age, etiology of cirrhosis, esophageal varices, ALP, GGT, ALB level, PLT counts, MELD score, and FIB-4 index. The associations between CIV levels and hepatic decompensation were statistically significant even after serum liver fibrosis markers were categorized according to the tertiles of serum levels in the whole series. In other words, this retrospective cohort study shows that serum liver fibrosis markers, especially in CIV, which were previously shown to be accurate markers of liver fibrosis, are also independent predictive factors for the liver decompensation that occur in cirrhotic patients with varying etiologies. Further prospective studies are required to confirm the role of serum liver fibrosis markers in these patients. To our knowledge, this is the largest study investigating the prognostic ability of serum liver fibrosis markers in a cohort of liver cirrhotic patients with varying etiologies. These findings reveal the stable prognostic role of CIV in compensated cirrhotic patients, which can identify high-risk patients early and will provide important insights into targeting ECM for the treatment of liver cirrhosis.

The etiology of cirrhosis of the patients is heterogeneous. The occurrence of decompensation differs considerably among the diseases. In addition, the triggers of decompensation are likely to differ among cirrhosis of different etiology. The heterogeneity must have caused confusion of the results. Thus, we also evaluated the predictive factors of decompensation in HBV-related cirrhosis because hepatitis B patients account for the highest proportion in this cohort. We found that after adjusting for potential confounders using a multivariable Cox regression model, higher LN and CIV levels were still found to be independently associated with hepatic decompensation. We assessed the three different decompensation events (ascites, esophageal bleeding, and HE) simultaneously [22, 23]. The triggers of these three events are likely to differ. Thus, we also evaluated the predictive factors for ascites due to the highest proportion of patients developing ascites in this cohort. We found that after adjusting for potential confounders using a multivariable Cox regression model, higher LN and CIV levels were also independently associated with ascites.

The evaluation of fibrosis provides considerable information and is highly valuable not only for.

the diagnosis but also for the prognosis and for the therapeutic decision. In recent years, due to the increasing need to easily and accurately stage liver fibrosis before treatment and monitor the progress of the disease, non-invasive markers of liver fibrosis have been extensively investigated [13, 24–27]. Biomarkers of liver fibrosis can be divided into direct and indirect markers [28]. Direct markers are fragments of the liver matrix components produced during the fibrotic process and the molecules represent the intensity of fibrogenesis or fibrinolysis such as HA, LN, CIV, and metalloproteinases [8]. The majority of earlier studies evaluating non-invasive fibrosis tests, were mainly cross-sectional with the aim of correlating levels of different types of simple and complex biomarkers or imaging with the stage of liver fibrosis [12, 29–32]. Only a small number of studies for these markers and their combination were designed to assess disease progression [33–35]. Nevertheless, these studies were performed in a low number of patients or focused on the value of HA.

Patients in the present study, who developed decompensation during follow-up had higher serum levels of HA, LN and CIV at baseline. It is well known that determination of ECM components may identify fibrosis of patients. Therefore, it is not surprising that higher HA, LN and CIV levels do indicate a more advanced fibrosis stage of the liver, which may logically have a higher likelihood of producing hepatic decompensation, which carry a poor prognosis. Our findings are in keeping with that of a previous study, which has shown that serum LN and HA, especially in LN, can be used as prognostic markers in addition to the CPS criteria in liver cirrhosis [35]. Nevertheless, the study was performed in a low number of patients and did not provide information on the value of CIV. HA is a glucosamine glycan existing in connective tissue, which is synthesized by mesenchymal cells and almost completely cleared by hepatic sinusoidal endothelial cells (about 1% is excreted through the kidney) [15]. HA has been extensively investigated as a simple non-invasive marker of fibrosis, with studies showing that it correlates with the degree of liver fibrosis [14, 35–37]. LN is synthesized by hepatocytes and sinusoidal cells, and is one of the main glycoproteins of basement membrane [38]. It has been reported that the serum fibrosis indices including LN can reflect the activity of liver fibrosis to a certain extent [39, 40]. CIV is an important component of normal ECM. Unlike type I and III collagen (partly processed proteolytically), CIV remains intact in the matrix and therefore serum components of CIV are considered to mainly reflect matrix degradation [41]. Serum CIV levels have previously been shown to correlate positively with the degree of hepatic fibrosis [12, 30, 31, 42]. Qi et al. suggested that CIV, LN, and HA levels were significantly associated with the severity of liver dysfunction, but might be inappropriate for the prognostic assessment of liver cirrhosis [43], which was inconsistent with our study. This may be due to the differences of the number of cases and the characteristics of patients.

Assessment of the risk of severe complications of cirrhotic patients has important clinical and therapeutical significance. The most widely used prognostic assessment in patients with liver cirrhosis is based on CPS and MELD score [2, 16]. The advantage of our study is the well-defined patient population of compensated cirrhosis with low MELD score. It is not easy to classify and identify patients who are likely to decompensate early. Therefore, defining predictors of decompensation is important for this group of patients. Moreover, the distribution range of the CPS and MELD score in compensated patients are much narrower than in decompensated patients because these patients often have normal TBIL, ALB, PT, INR, and Cr levels, and their increases only in presence of decompensation, whereas the LN and CIV levels, especially CIV, provide more information independent of MELD score for predicting liver decompensation due to their wider distribution. An important observation of our study was that patients with similar baseline MELD score had significant differences in LN and CIV levels. However, the trend for LN was not significant when it was categorized according to the tertiles of serum levels. In view of the results of our study, CIV appeared to be reliable biomarkers of disease progression and liver decompensation in patients with cirrhosis.

Our study argues for the fact that higher serum liver fibrosis markers levels should be recognized as major contributors which affects the occurrence of liver-related complications in patients with compensated cirrhosis. We do propose that patients with compensated cirrhosis should have a baseline tests of serum liver fibrosis markers and those with higher levels should be followed up more rigorously. Serum liver fibrosis markers are valuable, low-cost and easily available non-invasive predictors of liver decompensation in cirrhotic patients with varying etiologies. The implementation of the serum liver fibrosis markers tests in clinical routine could open up new strategies and allow individualized patient care. We also demonstrated that etiology of alcoholic cirrhosis, esophageal varices, older age, higher GGT, and lower PLT counts were independently associated with an increase in decompensation, which corroborate several prior publications [15, 44–47]. Therefore, we think that patients with alcoholic cirrhosis, esophageal varices, older age, higher GGT, and lower PLT counts at baseline were more likely to develop liver-related complications.

We recognized several limitations of our study. A potential limitation of the current study is that this was a retrospective analysis although all data was prospectively collected. Moreover, repeat data of serum liver fibrosis markers were not available in this cohort of patients, which reduced our ability to reliably determine the influence of changes in their levels on hepatic decompensation. This may require a large-scale prospective study with a series of measurements of serum liver fibrosis markers. Finally, patients from a longer distance do often miss their follow-up because they think they are keeping relatively well. Despite these limitations, as far as we know, this is the largest study investigating the prognostic ability of serum liver fibrosis markers in a cohort of cirrhotic patients with varying etiologies.

## Conclusion

In conclusion, this retrospective cohort study shows that serum liver fibrosis markers, especially in CIV, which were previously shown to be accurate markers of liver fibrosis, are also independent predictive factors for the liver decompensation that occur in cirrhotic patients with varying etiologies. Further prospective studies are required to confirm the role of serum liver fibrosis markers in these patients.

## Data Availability

The datasets used and/or analysed during the current study available from the corresponding author on reasonable request.

## Abbreviations

ALB: Albumin
ALT: Alanine aminotransferase
ALP: Alkaline phosphatase
AST: Aspartate aminotransferase
APRI: AST-to-PLT ratio index
CI: Confidence interval
CIV: Collagen IV
CPS: Child-Pugh score
Cr: Creatinine
ECM: Excessive deposition of extracellular matrix
FIB-4: Fibrosis-4 score
GGT: Gamma-glutamyl transpeptidase
HA: Hyaluronic acid
HE: Hepatic encephalopathy
HR: Hazard ratio
INR: International normalized ratio
LN: Laminin
MELD: Model for end-stage liver disease
PIIINP: N-terminal propeptide of type III collagen
PLT: Platelet
TBIL: Total bilirubin
